# Biomarkers of Survival in Patients with Colorectal Liver Metastases Treated with Percutaneous Microwave Ablation

**DOI:** 10.3390/cancers17071112

**Published:** 2025-03-26

**Authors:** Jakub Franke, Grzegorz Rosiak, Krzysztof Milczarek, Dariusz Konecki, Emilia Wnuk, Andrzej Cieszanowski

**Affiliations:** II Department of Radiology, Medical University of Warsaw, Banacha 1a, 02-097 Warsaw, Poland; jakub.franke@uckwum.pl (J.F.); krzys-km@wp.pl (K.M.); dariusz.konecki@uckwum.pl (D.K.); emilia.wnuk@wum.edu.pl (E.W.); andrzej.cieszanowski@wum.edu.pl (A.C.)

**Keywords:** liver lesion microwave ablation, post ablation survival, survival predictor, ablation biomarker, blood-derived survival predictor, locoregional treatment predictor

## Abstract

**Background/Objectives**: To evaluate the prognostic value of easily obtainable biomarkers for patients undergoing percutaneous microwave ablation (MWA) for colorectal liver metastases (CLMs). Prior studies showed that simple biomarkers, such as the lymphocyte-to-monocyte ratio (LMR), albumin-to-globulin ratio (AGR), platelet-to-lymphocyte ratio (PLR), and neutrophil-to-lymphocyte ratio (NLR), as well as cancer-specific markers, like carcinoembryonic antigen (CEA), might have a prognostic role in various malignancies; however, none of these were assessed in patients undergoing MWA for CLMs. **Methods**: Based on the simple laboratory results, which were determined prior to the ablation, several biomarkers, including the LMR, AGR, PLR, and NLR, were calculated. The log-rank test’s optimal cutoff points for continuous variables were determined. Subsequently, univariable and multivariable Cox regression models were utilized to determine the association between various features and overall survival (OS). **Results**: This study included 57 CLM patients with a mean age of 63 ± 12.5 years at the time of ablation with a mean follow up of 30.9 months. The univariable model demonstrated that a high level of CEA (cutoff: 29.1 ng/mL; HR: 3.70) and a high LMR (cutoff: 5.32; HR: 4.05) were related to worse OS, whereas a high NLR (cutoff: 2.05; HR: 0.31) and primary left-sided colon cancer (HR: 0.36) were positive prognostic factors. The multivariable regression model confirmed these findings, with the exception of the LMR, which was no longer significantly associated with OS. **Conclusions**: This study demonstrates the feasibility of overall survival prediction and thus patient stratification based on easily obtainable biomarkers and clinicopathological features in CLM patients undergoing MWA.

## Simple Summary

According to international guidelines, thermal ablation and surgery are the two main radical treatment possibilities for colorectal liver metastases. The aim of this study was to assess the prognostic value of simple laboratory-based biomarkers in patients undergoing microwave ablation for colorectal liver metastases. In a cohort of 57 patients, with a mean follow-up time of 30.9 months, higher levels of carcinoembryonic antigen and lymphocyte-to-monocyte ratio were linked to worse survival, while higher neutrophil-to-lymphocyte ratio levels and left-sided primary colon cancer were positive prognostic factors. A multivariable analysis confirmed most of the findings, except the lymphocyte-to-monocyte ratio’s significance.

## 1. Introduction

Due to the gradual buildup of evidence showing comparable efficacy to surgery in terms of the overall survival (OS) rates in liver tumor ablation, locoregional treatment has become one of the main treatment options for patients with both primary and metastatic liver cancer [1,2]. Radiofrequency ablation (RFA) has been the primary ablative modality for many years. With the advent of microwave ablation (MWA), most of the RFA limitations have been overcome. MWA can create larger ablation zones, the application time is shorter, and most significantly, it is suitable for lesions in close vicinity to vessels thanks to the lack of a ’heat sink effect’ when compared with RFA [3]. Currently, thermal ablation and surgery are the two main radical treatment possibilities for colorectal liver metastases (CLMs). According to many recommendations, they should be considered complementary methods depending on the size and location of the lesions, the patient’s comorbidities, and their preferences. According to the European Society for Medical Oncology (ESMO) guidelines, surgery or locoregional therapy is recommended as long as it is possible to eradicate the primary and oligometastatic disease. Similarly, current National Comprehensive Cancer Network (NCCN) guidelines for colorectal cancer state that locoregional treatment may be considered alone or complementary to surgery in case of the feasibility of an A0 ablation, which is defined as an ablation zone with margins over 10 mm [1,2].

Since the very beginning of ablative procedures, researchers have been looking for the predictive factors for local progression as well as overall survival, mainly focusing on the number and size of the lesions or the ablative margin [4,5,6,7]. Moreover, there have been studies trying to establish some readily available biomarkers, such as the lymphocyte-to-monocyte ratio (LMR), albumin-to-globulin ratio (AGR), platelet-to-lymphocyte ratio (PLR), neutrophile-to-lymphocyte ratio (NLR), and levels of cancer-specific markers, i.e., the carcinoembryonic antigen (CEA) and α-fetoprotein (AFP). The role of the aforementioned markers as independent predictive factors concerning survival has been demonstrated in patients with several malignancies [8,9,10,11]. However, most of these studies investigated patients treated with nonablative techniques. There are a few papers investigating biomarkers as OS predictors in patients treated with ablation, although a majority of them are based on cohorts with hepatocellular carcinoma treated with RFA [12,13,14]. The papers focusing on biomarkers in colorectal cancer patients undergoing ablation are limited [15,16,17]. To our knowledge, no study has investigated the LMR, PLR, NLR, AGR, and CEA in one cohort with colorectal liver metastases (CLMs) treated with MWA under CT guidance. Considering that colorectal cancer is one of the most commonly occurring malignancies, with the liver being the most common site of metastases, there seems to be a niche that was not thoroughly researched. Therefore, this study aims to provide a deeper insight into various clinicopathological predictors of survival.

## 2. Materials and Methods

This study included colorectal cancer patients with liver metastases who had been qualified for MWA by a multidisciplinary tumor board. The eligibility criteria for MWA were as follows: no extrahepatic disease, a platelet count > 50,000/mm^3^, and an international normalized ratio < 1.5. The ablation procedures were performed under CT (Aquillon Toshiba, Otawara, Japan) guidance by three interventional radiologists with at least six years of ablative experience. All procedures were performed under general anesthesia. The Solero MWA applicator system (AngioDynamics BV, Amsterdam, The Netherlands) was used. The number of ablative sessions and application parameters was based on the lesion location and size to obtain an oncologic margin of at least 5 mm but preferably 10 mm. The technical success of the procedure was confirmed with a control triple-phase contrast-enhanced CT study performed at the end of the procedure. In case any unablated tumor was visible while the control study was performed during the procedure, the ablation needle was reintroduced and an additional ablation session was performed during the same procedure to destroy the viable tumor tissue and achieve complete local control. Subsequently, all patients underwent a routine follow-up six weeks after the procedure to confirm the radicality of the ablation, and then every three months for a year to detect a possible early recurrence, which could be reablated. Further, a follow-up scheme, typically an MRI scan every 6 months, was implemented according to the referring physician’s discretion.

**Figure 1:**
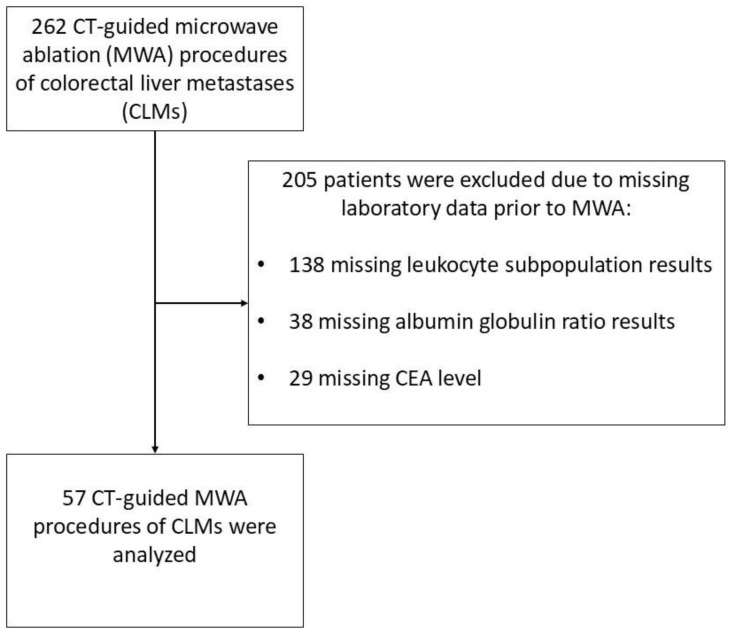
Patient selection flowchart of a study group consisting of patients with colorectal cancer liver metastases treated with microwave ablation.

Between 2017 and 2022, 262 patients underwent a CLM ablation using MWA. However, out of the 262 patients who were treated, 57 patients had a complete blood count with defined leukocyte subpopulations, CEA value, and an albumin level tested prior to the procedure. Only data from patients who met these inclusion criteria were used for statistical analysis (Figure 1).

The CEA density (ng/mL·mm^3^) was calculated based on the total CEA serum level before ablation, divided by the total volume of the metastatic lesions located in the liver, similar to the method used by Hou et al. to calculate the CEA density in patients with colorectal lung metastases [16].

All the statistical analyses were performed using the statistical libraries available for Python version 3.9 and R version 4.2.3 (R Foundation for Statistical Computing, www.r-project.org). Right censoring was used when no event occurred during this study. Therefore the length of follow-up was defined as the time from ablation to death or the last available follow-up data. The optimal cutoff points for potential continuous predictors were calculated using a log-rank test. The value of a variable with the highest log-rank statistical value was accepted as a cutoff point. Furthermore, the variables were assessed using Cox regression models with a statistical significance level of a *p*-value < 0.05. A multivariable Cox regression model was created based on the variables that achieved significance in a univariable analysis. Furthermore, to avoid the bias resulting from possible collinearity all the variables in the multivariable analysis were checked using a variance inflation factor (VIF) analysis. For each variable in the model, the VIF was below 2 indicating that multicollinearity is not a significant issue in this model.

The Bioethical Committee accepted retrospective nature of this study (AKBE 6/2024). All procedures were performed in accordance with the declaration of Helsinki, as revised in 2013 and written consent for procedures were obtained from the patients.

## 3. Results

A total of 30 males and 27 females were included in this study with a mean age of 63 ± 12.5 years (29.5–87.9 years) at the time of ablation. In the majority of cases there was a single metastatic lesion (44/57) and only a few patients had more than two lesions (6/57). The mean diameter of the lesion was 20 ± 10.4 mm (5–59 mm), with all the lesions except one being below 50 mm. Moreover, the majority of patients (43/57) had lesions of a diameter below 30 mm. The mean follow-up period was 30.9 months ± 15.1 months (6.7–68.8 months).

A log-rank analysis was used to determine the most significant cutoff values for the biomarkers, LMR, AGR, PLR, NLR, CEA, and the CEA density (Table 1).

**Figure 2:**
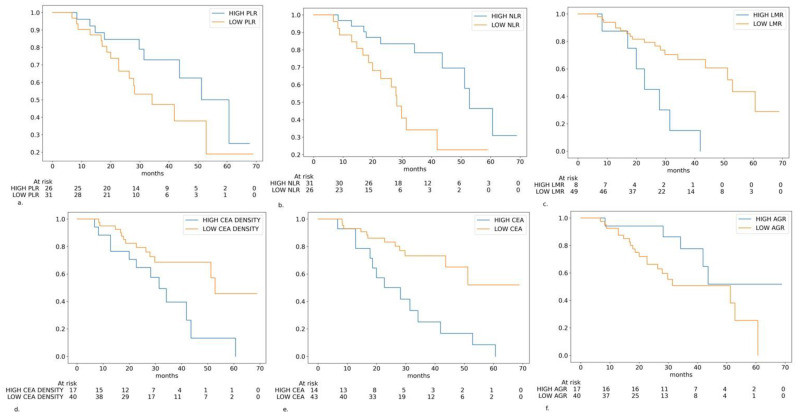
Kaplan–Meier curves in the different subgroups of patients based on the biomarkers levels: (**a**) platelet-to-lymphocyte ratio (PLR) level (cutoff: 122.7); (**b**) neutrophile-to-lymphocyte ratio (NLR) level (cutoff: 2.05); (**c**) lymphocyte-to-monocyte ratio (LMR) level (cutoff: 5.32); (**d**) carcinoembryonic antigen (CEA) density (cutoff: 0.00046 ng/mL·mm^3^); (**e**) CEA serum level (cutoff: 29.1 ng/mL); (**f**) albumin-to-globulin ratio (AGR) level (cutoff: 1.81).

In the univariable statistical analysis, the LMR, CEA, CEA-density, NLR, and primary tumor sidedness were statistically significant predictors of OS. The AGR and PLR were borderline non-significant. Moreover, several other features were not associated with OS (Table 2, Figure 2).

From the statistically significant variables, high levels of the NLR and primary left-sided tumor were protective prognostic markers, whereas high levels of the LMR, CEA and CEA-density were associated with worse prognoses. The strongest impact was demonstrated by the LMR (HR: 4.05) and CEA (HR: 3.70).

A multivariable Cox regression model using features that showed statistical significance in univariable analysis was created. In this model all the variables except the LMR remained associated with OS (Table 3).

## 4. Discussion

The role and importance of the locoregional treatment of liver metastases originating from CRC as well as other gastrointestinal (GI) tumors keeps on increasing. The ablation of CLMs in case of small lesions is supported by international guidelines [1,2]. A recent randomized clinical trial demonstrated the non-inferiority of the CLMs ablation in the context of OS with a favorable safety profile when compared to surgical resection [18]. Ablation is also included as the treatment option in patients with oligometastatic neuroendocrine tumors (NETs) in case of the earlier resection of the primary tumor, as well as palliative treatment to relieve hormonal symptoms in the advanced stages [19,20,21]. In pancreatic cancer, the ablation of metachronous hepatic lesions is not included in the international guidelines; however, there are papers demonstrating decent results in oligometastatic patients treated with ablation or ablation combined with chemotherapy [22,23,24]. Nonetheless, the number of evidence supporting the role of locoregional treatment in the setting of metachronous metastatic pancreatic cancer is limited and probably should be studied more extensively. Another intriguing concept in the treatment of metastatic GI tumors is the management of patients with an advanced disease and liver-only metastases. There is increasing evidence that a highly specific subset of patients might benefit from surgical treatment of primary tumors and metastases, both in cases of NET as well as pancreatic cancer [25,26,27]. With further studies on this topic, one can expect that locoregional therapy will also have its role in case of unresectable but ablatable hepatic metastases in this highly selected group of patients. Having in mind the increasing role of MWA in the management of hepatic metastasis, different origin studies investigating the detailed predictors of OS are needed.

This study investigates the role of the various clinicopathological factors of OS predictors in patients undergoing the microwave ablation of liver metastatic lesions, mainly focusing on easily available biomarkers based on peripheral blood tests. The rationale of using biomarkers is based on the fact that the systemic inflammatory response was demonstrated to be a vital part of cancer initiation, growth, metastasis, and treatment response. Lymphocytes play a crucial role in the cancer immune response via the infiltration of the cancer microenvironment, recognition of neoantigens, and further stimulation of cancer-specific response. On the contrary, neutrophils and monocytes stimulate angiogenesis and suppress the immune response, thus leading to tumorigenesis and tumor promotion [28,29]. Therefore, the multiple markers based on the peripheral blood cell counts reflect the state of the host’s anticancer immune response.

According to our knowledge, no study assessed the LMR, NLR, PLR, AGR, and CEA in a single cohort of patients with CLMs treated with MWA. There are few studies investigating these biomarkers in patients treated with RFA. Although these ablative modalities have some practical differences, they are both heat-based technologies. The aforementioned biomarkers are continuous variables with no widely accepted cutoff value, which can be recognized as abnormal. Therefore, the log-rank test’s most significant cutoff points for each biomarker in the study population were determined. This analysis of the biomarkers demonstrated that the LMR, NLR, CEA and CEA density were associated with OS. Moreover, the analyzed biomarkers had a high impact on OS as the HR ranged from 0.31 to 4.05 in case of the strongest protective and negative factors, respectively. To further validate the results, the multivariable analysis was performed and to limit the risk of false positive findings all the variables in the model were checked for collinearity using the variance inflation factor. The NLR and CEA biomarkers remained significant predictors of OS with similar results to the univariable analysis of HR (0.29 and 4.1, respectively). The observed impact of both the NLR and CEA is really high, and although in accordance with other studies signifying the importance of the host immune system, as well as the overall tumor burden in predicting OS, the degree of the effect in this cohort is intriguing and probably requires further investigation with larger studies to better control potential confounding factors. However, in the multivariable Cox regression analysis, the LMR did not maintain its association with OS. One of the reasons for such a finding might be the fact that there is no widely accepted and optimal cutoff value for the LMR to be used in survival prediction as its cutoff points used in studies vary widely, and in some studies a too high cutoff resulted in the lack of the LMR’s significance in the subgroup analysis [30,31,32]. Moreover the effect of the LMR on OS might have been diminished when paired with other possibly stronger survival predictors in multivariable analysis. Similar findings in which a certain biomarker was significant in univariate analysis but the significance did not persist in multivariate analysis can be found in other studies [12,15,33]. Both the CEA levels as well as CEA density can be interpreted as the biomarkers of the tumor burden and, in contrast to LMR, NLR or PLR, are not directly related to the patient’s immune status. In this study, the high CEA level (>29.1 ng/mL) was one of the most significant predictors in the univariate analysis (HR: 3.70; *p*: 0.001) and the most significant predictor in the multivariate model (HR: 4.10; *p*: 0.003).

In general, the findings are in accordance with other studies based on nonablative treatment modalities, which report the LMR, NLR, and CEA as significant survival predictors [10,32,33,34]. It is worth noting that although in this study the PLR and AGR were not significant OS predictors, the obtained *p*-value was close to the significance level, and the hazard ratios were in accordance with the papers reporting the association of these biomarkers and OS [8,13,35].

Zhang et al. reported worse OS and disease-free survival (DFS) in patients with higher NLR after the RFA treatment of CLMs. The NLR threshold of > 5.0 was used as a cutoff point [36]. Chang et al. determined an NLR level of > 2.5 to be linked with worse DFS [37]. Moreover, a higher NLR was determined to be a poor prognostic factor in various other studies, including patients treated with nonablative modalities. However, the cutoff values used in these studies ranged from 1.9 to 7.26 [38]. Quite significant differences in the threshold levels reported by various papers can be possibly attributed to the character of the tumor microenvironment and the interaction between the tumor and host’s immune system. Tumor cells are known to secrete various cytokines and chemokines, which might affect the infiltration of the tumor microenvironment as well as the counts of leukocyte subgroups [28,29]. The accordance in reporting higher NLR levels as a poor prognostic factor in cohorts treated with different modalities probably highlights the importance of the host’s immune system anticancer response as the major factor determining the patient’s prognosis irrespective of the treatment modality.

Facciorusso et al. investigated many possible biomarkers, including absolute counts of leukocyte subgroups, NLRs and LMRs in patients treated with RFA. In their study, the NLR with a threshold of 2.1 and the LMR with a threshold of 3.96 predicted survival in univariable models. However, in multivariable regression, only the LMR remained significant [15]. Such findings are in accordance with this paper, in which both the LMR and NLR are predictors of survival in the univariable analysis (HR:4.05 and 0.31, respectively) with cutoff values of 5.32 and 2.01, respectively. However, in opposition to the paper by Facciorusso et al., the LMR was no longer significant in the multivariable regression model.

Contrary to the results of this study, the PLR and AGR have also been linked with patients’ survival in several papers. However, most of them were based on a series of patients treated with nonablative modalities [8,10,11]. Although not in a cohort of CLM patients but in a series based on HCC patients treated with ablation, Wang et al. reported the AGR, NLR, and PLR in the univariable analysis to be associated with survival. In their multivariable analysis, the AGR and PLR remained significant. Notably, the LMR lacked such an association [13].

The role of the CEA serum level as a survival predictor is not fully understood. In most papers, a higher CEA level was reported to be linked with worse OS in patients with CLMs. However, some reports demonstrate a lack of such a link [5,6,15,16].

Hou et al. published an interesting approach. In their paper, the CEA density, defined as the total CEA serum level divided by the total volume of lesions, was a better OS predictor than the total CEA serum level. These results are in accordance with the literature, both in case of worse OS in the high CEA subgroup as well as the cutoff point used to stratify patients into subgroups, which commonly ranges from 29 to 35 ng/mL [5,15].

The impact of the sidedness of the primary cancer on the OS is also not fully elucidated, with papers supporting both theses, not only in cases of surgical resection but also thermal ablation. For example, Makowiec et al. and Zhou et al. [39,40] reported a lack of association between the sidedness of the primaries and OS in the case of resection and MWA, respectively. Interestingly, Dijkstra et al. [41] published an impressive series comprised of 520 patients with a total of 2101 CLMs who were undergoing local treatment and reported no association between the primary tumor sidedness and OS or local tumor progression-free survival. However, a difference in distant progression-free survival was observed. On the other hand, papers showed better OS in left-sided primary surgical and thermal ablation treatment [42,43]. In a reported cohort, sidedness was a significant predictor of OS. Left-sided colon cancer patients had better OS when compared to right-sided colon cancer counterparts (HR: 0.36)

This study has some limitations that should be taken into consideration. Firstly, it is a retrospective analysis from a single institution. The procedures were performed by three different interventional radiologists. However, this limitation is partially overcome due to the similar experience of the operators and the verification of the ablation zone with a contrast-enhanced CT scan immediately after the procedure. When analyzing the CEA serum concentration, one should remember that extrahepatic disease might affect the levels of this marker and that other non-oncological conditions are capable of increasing the CEA serum levels. The patients, before the ablation, were screened for extrahepatic disease and disqualified if any was present. Therefore, any potential extrahepatic lesions were radiologically occult and thus probably unable to affect the CEA levels significantly. Moreover, in some cases in the cohort, MWA was not the only treatment modality that patients underwent; thus, the observed OS may be confounded by other therapies and not linked only to the effect of MWA.

Some of the aforementioned limitations can be considered at least partially overcome; nonetheless, to address these limitations, future research should ideally involve prospective, multi-institutional studies with larger cohorts and longer follow-up periods. Such studies would allow for better control over potential confounders and provide more robust evidence on the predictive value of these biomarkers and the degree of the effect they have. It is worth noting that this study has several strengths. According to our knowledge, there is no other study investigating such a range of biomarkers in a single cohort of patients with CLMs who are undergoing thermal ablation. Moreover, the majority of papers focusing on the studied biomarkers come from cohorts treated with RFA, with a scarcity of data from cohorts treated with MWA. Both RFA and MWA are heat-based ablative modalities; however, currently, MWA is more frequently used in the setting of CLMs, thus increasing the value and adequacy of the findings of this study. Additionally, the ease of measurement, cost-effectiveness, and prognostic value of the investigated biomarkers make them promising tools, which can be utilized independently of the clinical setting, available laboratory equipment or healthcare funding models. While none of the biomarkers analyzed, apart from CEA and the CEA density—LMR, NLR, PLR, AGR—are specific to CLM, their value stems from their ability to reflect the systemic inflammatory response and tumor burden, both of which have a well-established role in cancer progression and treatment outcomes. These results suggest that the host immune response as well as the tumor burden play a major role in the OS prediction of patients treated with MWA. These findings can help refine patient selection for MWA, identifying those who may benefit most from the procedure or those who might require closer follow-up.

The prospective validation of these findings in larger, multicenter cohorts could lead to the development of standardized prognostic models integrating these biomarkers with clinical and radiologic factors similar to other scores or nomograms, like the surgical clinical risk score (CRS) or modified ablation CRS [5,13,44].

## 5. Conclusions

This study provides a deeper insight in efforts to create more individualized predictive models of OS for CLM patients undergoing MWA and the majority of the results are in line with prior studies based on RFA cohorts with HCC or CRC lesions. However, there are some discrepancies between the studies, which demonstrate that the role of biomarkers remains unclear, and further, that preferably multi-institutional studies based on larger cohorts and longer follow-up times are desired to provide necessary data to eventually elucidate the significance of these factors. Furthermore, such data may facilitate the creation of more sophisticated models and nomograms, which would hopefully improve patient selection or follow-up schemes.

## Figures and Tables

**Table 1:** Analyzed blood biomarker characteristics in the study group of patients with colorectal cancer liver metastases treated with microwave ablation.

Biomarker	Mean ± SD	Range	Cutoff Value
LMR	3.44 ± 1.84	1.34–10.05	5.32
AGR	1.66 ± 0.32	0.80–2.60	1.81
PLR	131.09 ± 74.68	18.64–383.02	122.7
NLR	2.36 ± 1.39	0.79–8.29	2.05
CEA [ng/mL]	30.47 ± 59.97	0.32–323.00	29.1
CEA density [ng/mL·mm^3^]	0.0019 ± 0.01	0.000004–0.07	0.00046

Lymphocyte-to-monocyte ratio (LMR), albumin-to-globulin ratio (AGR), platelet-to-lymphocyte ratio (PLR), neutrophile-to-lymphocyte ratio (NLR), carcinoembryonic antigen (CEA).

**Table 2:** Results of the overall survival univariable analysis of the blood biomarkers and clinical features in the study cohort.

Variable	HR	HR (95% CI)	*p*-Value
LMR (reference > 5.32)	4.05	1.62–10.12	0.003 *
AGR (reference > 1.81)	0.38	0.14–1.03	0.058
PLR (reference > 122.7)	0.49	0.22–1.13	0.094
NLR (reference > 2.05)	0.31	0.13–0.72	0.007 *
CEA (reference > 29.1 ng/mL)	3.70	1.68–8.14	0.001 *
CEA density (reference > 0.00046 ng/mL·mm^3^)	2.55	1.16–5.62	0.020 *
Primary CRC location (reference left-sided)	0.36	0.15–0.85	0.019 *
Age	1.00	0.97–1.03	0.924
Gender (reference male)	1.34	0.60–2.96	0.472
Number of lesions	1.04	0.74–1.46	0.837
Diameter (reference > 30 mm)	1.07	0.46–2.50	0.875

* statistically significant (*p*-value < 0.05). The lymphocyte-to-monocyte ratio (LMR), albumin-to-globulin ratio (AGR), platelet-to-lymphocyte ratio (PLR), neutrophile-to-lymphocyte ratio (NLR), and carcinoembryonic antigen (CEA).

**Table 3:** Results of the overall survival multivariable analysis of the blood biomarkers and clinical features in the study cohort.

Variable	HR	HR (95% CI)	*p*-Value
LMR (reference > 5.32)	1.03	0.31–3.41	0.96
NLR (reference > 2.05)	0.29	0.11–0.82	0.018 *
CEA (reference > 29.1 ng/mL)	4.10	1.60–10.54	0.003 *
Primary CRC location (reference left-sided)	0.25	0.10–0.64	0.004 *

* Statistically significant (*p*-value < 0.05). The lymphocyte-to-monocyte ratio (LMR), neutrophile-to-lymphocyte ratio (NLR), carcinoembryonic antigen (CEA), and colorectal cancer (CRC).

## Data Availability

The original contributions presented in this study are included in the article. Further inquiries can be directed to the corresponding author.
